# Effectiveness of remotely delivered speech therapy in persons with Parkinson's disease – a randomised controlled trial

**DOI:** 10.1016/j.eclinm.2024.102823

**Published:** 2024-09-11

**Authors:** Janna J.L. Maas, Nienke M. de Vries, Joanna IntHout, Bastiaan R. Bloem, Johanna G. Kalf

**Affiliations:** a Radboud University Medical Center, Donders Institute for Brain, Cognition and Behaviour, Department of Neurology, Center of Expertise for Parkinson & Movement Disorders, Nijmegen, the Netherlands; b Radboud University Medical Center, IQ Health; Nijmegen, the Netherlands; c Radboud University Medical Center, Donders Institute for Brain, Cognition and Behaviour, Department of Rehabilitation, Nijmegen, the Netherlands

**Keywords:** Parkinson's disease, Speech therapy, Telemedicine, Randomised clinical trial

## Abstract

**Background:**

Increasing evidence supports the merits of speech therapy in Parkinson's disease, but the current practice of multiple in-house treatments is demanding for patients. We therefore assessed the effectiveness of remotely delivered and personalised speech therapy on improving quality of life and speech quality in persons with Parkinson's disease.

**Methods:**

We performed a single blinded randomised controlled trial (the PERSPECTIVE study), comparing 8 weeks of personalised remote speech therapy to no intervention (waiting list design). Patients with reduced speech intelligibility were included, regardless of disease stage or dysarthria severity. Patients were assigned randomly (1:1) to the intervention or control group. Measurements took place at baseline and after 8 weeks (both groups), and after 32 weeks (intervention group only). Patients were treated remotely by 20 experienced speech therapists. The primary outcome was disease-related quality of life at 8 weeks, assessed with the Parkinson's Disease Questionnaire 39 (PDQ-39). Data were analysed using analysis of covariance based on the intention-to-treat principle. This trial is registered in ClinicalTrials.gov, NCT03963388.

**Findings:**

Between March 1, 2019, and March 27, 2021, 214 patients were enrolled in the intervention group (n = 109) or control group (n = 105). At the primary timepoint, the adjusted mean difference in PDQ-39 was −2.0 in favour of the intervention group (95% CI −4.0 to 0.1); p = 0.056). The intervention group scored better on the communication index score of the PDQ-39 (post hoc analysis), with an adjusted mean difference of −5.3 (95% CI −9.4 to −1.2; p = 0.011). We found no between-group differences on any other PDQ-39 domain. Follow-up measurements showed a significant reduction of the PDQ-39 compared to the primary timepoint with a difference of 2.40 (95% CI 0.77–4.02; p = 0.004).

**Interpretation:**

Personalised remote speech therapy improved communication-related quality of life, but not overall quality of life.

**Funding:**

10.13039/100000864Michael J. Fox Foundation, Gatsby Foundation, and 10.13039/100016036Health∼Holland.

**Research in context**

**Evidence before this study**

Two systematic reviews concluded that speech therapy may improve speech impairments in Parkinson's disease, but the quality of the evidence remains limited. A large, well-designed randomised controlled trial remains needed to demonstrate the effectiveness of speech therapy, including outcome measures relevant to people with PD and a follow-up of at least six months to determine the duration of improvement. Earlier work mostly included participants with mild-moderate Parkinson's disease, and more work is needed to demonstrate the efficacy of speech therapy for individuals with a wider range of disease severity. Telerehabilitation can be considered to improve accessibility to speech therapy.

**Added value of this study**

Here, we addressed the above mentioned knowledge gaps by performing a large randomised controlled trial with a long-term follow-up, and with outcomes relevant to people with PD. We included patients regardless of disease stage, offering insights into the effectiveness of speech therapy across patients with different disease severity.

**Implications of all the available evidence**

The combined evidence suggests that speech therapy can improve communication-related quality of life of people with PD, and also speech quality and functional communication. Future work should focus on strategies to implement personalised speech therapy for people with Parkinson's disease across all disease stages.

## Introduction

Up to 70% of persons with Parkinson's disease (PD) may encounter problems in speech intelligibility due to hypokinetic dysarthria,^1^ affecting social interaction and quality of life. Hypokinetic speech is characterized by a soft and monotonous voice and small articulation movements, which worsens when tired.^2^ Since pharmacological treatment offers limited improvement,^3^ speech therapy is the preferred option to improve speech quality and intelligibility. This is now deemed to be an integral part of comprehensive care for PD across all disease stages.^4^ Recent systematic reviews^5^, ^6^, ^7^ conclude that the evidence for the effectiveness of speech therapy in PD is increasing. However, more research is needed to explore its full potential since previous studies had certain limitations, including small samples or short follow-up periods. Also, the outcome measures used in previous studies were suboptimal because of their limited clinical relevance. Finally, generalizability was restricted because mostly participants with mild to moderate PD were included (i.e. excluding those with more advanced disease stages).^5^, ^6^, ^7^

Current speech treatments (Lee Silverman voice treatment (LSVT),^8^^,^^9^ Pitch Limiting Voice Treatment (PLVT),^9^^,^^10^ and SPEAK OUT!^10^) generally focus on speaking louder to overcome the hypokinetic speech. Intensive training in these programs is imperative for patients to gain and preserve a better intelligibility. This requires a highly intensive treatment program, typically involving in-house treatment sessions three to four times a week, for a duration of on average four weeks. However, such a high-intensity approach is less feasible for patients with severe PD, so a more personalised and convenient approach is needed. In that regard, telerehabilitation has become an attractive way to offer treatments remotely, into the home of affected individuals. This is particularly attractive for more severely affected patients with difficulties traveling and for patients living in loosely populated areas where physical access to specialised care is challenging. The COVID-19 pandemic has shown that telerehabilitation can be a suitable solution for delivering treatments during times of lockdown.^11^ Treatment in the comfort of the patients’ own home makes it easier to achieve a high frequency of training. Previous studies already suggested a non-inferiority of online speech therapy and an acceptable feasibility of other speech-related activities such as therapeutic singing in small groups of PD patients, as compared to the traditional in-house treatment programs.^12^, ^13^, ^14^, ^15^

Here, we report the results of the PERSPECTIVE study (PERsonalised SPEech therapy for aCTIVE conversation), a Randomized Controlled Trial (RCT) evaluating the effectiveness of a remotely delivered speech therapy program in persons with PD who experienced a reduced speech intelligibility, but irrespective of dysarthria severity and across all disease stages. We expected speech therapy to improve both quality of life and speech quality in patients across all disease stages. Moreover, we hypothesised that the improvements would be maintained at the six-month follow-up.

## Methods

We conducted a single blinded RCT, comparing remotely delivered speech therapy with a waiting-list control group. The study protocol, approved by the medical ethical committee of Arnhem-Nijmegen (NL67867.091.18), has been detailed previously.^16^

We recruited patients diagnosed with PD according to the Movement Disorder Society criteria,^17^ in all disease stages. Potential participants were asked to participate by their own speech-language therapist (SLT) at the time of initial referral, or they volunteered to participate via the Dutch online recruitment platform ParkinsonNext (www.parkinsonnext.nl) which matches patients to suitable research projects. Interested patients received an information letter with detailed information about the study. To provide the patients enough time to consider participation, the research team called the patients seven days after having received the information letter, to verify their willingness to participate and to check for eligibility. When a participant was willing and eligible, the baseline measurement (T0) was scheduled. Eligibility criteria were: (1) reduced intelligibility that negatively impacted daily communication, as indicated by the patient or informal caregiver(s); (2) a desire to improve their speech; and (3) willingness and ability to receive online treatment. Exclusion criteria were: (1) recently (<1 year) having received speech therapy; (2) voice or speech problems due to other causes; (3) communication difficulties based on language problems without predominantly reduced intelligibility; and (4) (technical) inability to receive online treatment. When willing and eligible, a primary informal caregiver was also asked to participate. Both patient and caregiver signed informed consent in twofold before the start of the baseline measurement.

For detailed descriptions of the procedures and outcomes, we refer to a previous publication of the study design.^16^

### Randomisation and masking

After the baseline visit, patients were randomised to either the intervention or the waiting list control group in a 1:1 manner by means of a computerized validated variable block randomization model with automatically generated block sizes of 4, 6, and 8. Stratification took place for sex, current age (<46 years, 47–55 years, 56–65 years, >66 years), speech language therapist, and Hoehn & Yahr stage. Randomisation was performed by a researcher who was not involved in any of the measurements, and using the electronic data capture system Castor EDC (Amsterdam, NL). This researcher subsequently informed both the therapist and patient about group allocation, keeping the researchers who performed assessments and analyses blinded to treatment allocation. Allocation of participants was made invisible for assessors in Castor EDC. Patients and therapists could not be masked, but were instructed not to talk about the treatment allocation with the assessors. The assessors kept track of what presumption they had about group allocation for every participant (intervention group, control group, or no idea).

### Procedures

**Fig. 1:**
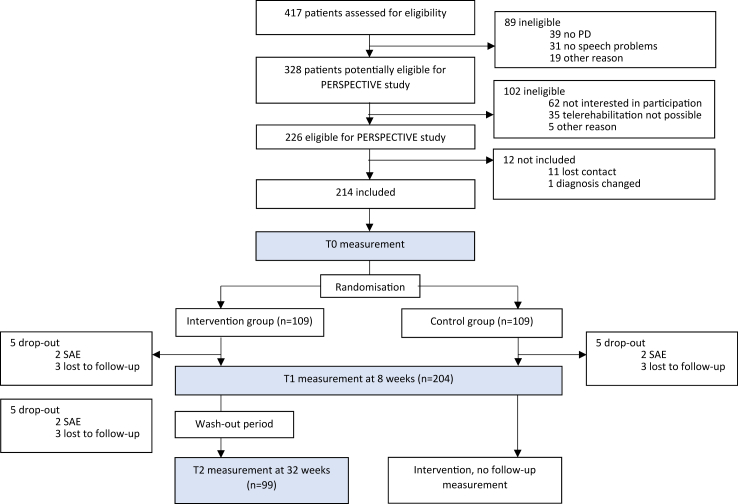
Trial profile.

Measurements took place in the patients’ homes at three time points: T0 measurement (baseline), T1 measurement at 8 weeks after baseline (primary endpoint), and T2 measurement at 32 weeks after baseline (secondary endpoint, only for the intervention group). If the patient was allocated to the intervention group, treatment started within a week. If the patient was allocated to the control group, treatment started after the second measurement (T1), as shown in the flowchart (Fig. 1). No T2 measurements took place for the control group. We decided not to include this, because in this trial, having insights into whether the effects of speech therapy would be maintained in the long term was only a secondary question, and we considered it more important to optimise our trial design for the primary research question that was focused on T1. To optimise compliance for the control group, we wanted to keep the waiting list brief, which motivated us to offer them the delayed treatment immediately after T1, instead of after T2.

Data were collected by three assessors. Questionnaires were completed online by the patient within three days after each visit. At the end of the baseline assessment, patients received an explanation on how the remote treatment would take place.

### Intervention

The intervention was delivered by 20 speech-language therapists (SLTs) throughout the Netherlands. All therapists participated in the Dutch ParkinsonNet, which is a nationwide healthcare approach of regional multidisciplinary networks of healthcare professionals specialised in PD, aiming to provide high-quality care for patients with PD.^18^ Therapists were selected based on their experience and caseload (minimum of one new PD patient per month for a full treatment) to ensure treatment quality and to ascertain a stable inclusion. The treatment delivery was planned for a maximum period of 8 weeks. The exact number of sessions depended on the complexity of goals and capacity of the patient. Between T1 and T2, participants in the intervention group did not receive any further speech therapy.^19^ The PERSPECTIVE treatment approach combines three elements: (1) treatment delivery in the patient's home using telerehabilitation, as a replacement for in-house therapy; (2) personalised treatment approach; and (3) consistent use of a feedback-app for smartphone or tablet that delivers real-time visual feedback.

1. Highly experienced therapists who deliver online treatment

In addition to the initial three-day training course by the Dutch ParkinsonNet, the SLTs participating in the study followed an extra two-day training during which they were informed about the study procedures and the PERSPECTIVE intervention protocol, including delivering online treatment. Delivery of the online treatment was done using a reliable, certified and secured online platform, which is easy to use without extra costs for patients (software provided by Zaurus B.V.). To guarantee compliance and treatment quality, participating SLTs were supported during the study by a coach (one of two expert SLTs). Coaching took place during a full treatment session in week 1, 3 and 5 for every patient via a group video call with the patient, the SLT and the coach, creating the opportunity to provide live detailed feedback to the patient and the SLT when needed.

2. Personalised treatment

The treatment is based on the Dutch Guidelines for Speech-Language Therapy in Parkinson's Disease^20^ and the Pitch Limiting Voice treatment protocol.^21^ The techniques to improve intelligibility were personalised, based on the patient's individual treatment goal and ability to receive intensive treatment. The optimal treatment goal of PLVT is to independently speak intelligibly in all communication situations in order to regain social participation. PLVT is provided 3–4 times per week for 30 min, with a maximum treatment trajectory of 8 weeks. Patients also perform daily home-based exercises. If the goal of independently speaking intelligibly is not realistic for the patient, then optimal high-intensive treatment is still available, but a lower goal is set, namely speaking intelligibly with support from a caregiver or conversation partner. The treatment intensity, frequency, and goals were adjusted on a personal basis, based on the insights of the speech therapist.

Some patients might experience limitations such as cognitive decline or severe fatigue, making intensive treatment and the goal of independently speaking intelligibly not feasible. In these circumstanced, patients received less intensive treatment (1–2 times a week for 30 min), thereby accepting more modest treatment results compared to high intensive treatment. Also, if present, the caregiver was explicitly involved and, when necessary, instructed on how to support the patient in performing exercises or providing trained cues.

3. Support by feedback-app

To provide PD patients with specific feedback on how to improve their speech, we developed a dedicated app that delivers real-time visual feedback on speech loudness and pitch: the Voice Trainer.^19^ After the SLT sets frequency (Hz) and loudness (dB) levels, the Voice trainer provides intuitive feedback using a circle on the screen of the device. If the circle is green and large, this means the speech is loud enough. Does it become red and small, the user speaks too softly. The placement of the circle reflects pitch: when the circle is placed up in the screen, the pitch is too high. Ideally, the circle is placed in the middle or slightly under the middle of the screen. The interface of the Voice trainer is available in Supplement 1. The app supports patients and therapists to practice and correctly maintain voice loudness and pitch, during exercises as well as beyond the therapy sessions. Using an online dashboard connected to the Voice trainer of the patient, SLTs were not only able to see how many times the Voice trainer had been used by the patient, but also how well the patient performed regarding percentages of correct loudness and pitch. This allowed the SLT to keep track of the daily home exercises. To use the Voice Trainer and the online platform for video conferencing at the same time, patients needed two devices (preferably a smartphone and a tablet or computer).^16^ Patients who did not have a supporting device for online therapy could loan a tablet during study participation.

### Outcomes

#### Primary outcome measure

Our primary endpoint was quality of life using the summary index score of the Parkinson's Disease Questionnaire (PDQ-39) at 8 weeks follow-up (T1). The PDQ-39 is the most widely used PD-specific health-related quality of life questionnaire.^22^ A decrease of this score over time suggests improvement.

#### Secondary outcome measures (observer-rated)

Observer-rated secondary outcome measures were evaluation of speech and voice quality (speech tasks as described in the Radboud Dysarthria Assessment^23^; maximum repetition rate, maximum phonation duration, maximum pitch range, maximum loudness level, and dysarthria severity) and Acoustic Voice Quality Index^24^), speech intelligibility (Dutch intelligibility test—sentence level^25^), and swallowing speed (maximum swallowing speed^26^).

#### Secondary outcome measures (patient-rated)

Patient-rated outcome measures were voice-related complaints (Voice Handicap Index^27^), Parkinson-specific speech-related complaints (Radboud Oral Motor inventory for Parkinson's disease^28^), mood and anxiety (Hospital Anxiety and Depression Scale^29^), and health-related quality of life (EuroQol-5D^30^). Outcome measures for caregivers were caregiver burden (Zarit caregiver Burden Interview Short Form^31^), and caregiver reported Parkinson-specific speech-related complaints (Radboud Oral Motor inventory for Parkinson's disease, adapted to caregiver).

### Sample size

Based on a trial on the effectiveness of multidisciplinary care,^32^ we calculated the sample size based on our primary outcome with a conservative estimated PDQ-39 total score improvement of 2.5 points in the intervention group, and no difference in controls (assuming a shared SD of 5.8). This estimated improvement exceeds the minimally important difference of 1.6 points.^33^ A sample of 170 patients would be needed to show this expected difference. Allowing for 20% drop-out, we aimed to include a maximum of 215 patients.

### Statistical methods

#### Descriptive statistics

Depending on whether the outcomes were normally distributed (based on visual inspection of Q–Q plots, the presence of outliers, and whether there is a clinically relevant difference between the mean and median), means and standard deviations (SD) or median and interquartile range and frequencies are used to describe the outcome, demographics and baseline variables.

#### Analysis of effectiveness

Based on our hypothesis and design, two comparisons were made: at the primary endpoint (T1), we compared the mean PDQ-39 between the intervention group and the control group. At the secondary endpoint (T2), we identified the long-term effects of speech therapy in the intervention group after 24 weeks of follow-up. Statistical analyses were performed based on the intention-to-treat principle. For the primary endpoint, analyses of covariance (ANCOVA) was used with group allocation as fixed variable. Baseline values of the dependent variable, age at baseline, Hoehn & Yahr stage and disease duration served as covariates. For the PDQ-39 at T2, a paired sampled T-test was performed.

#### Additional analyses

To gain insights into the different domains of the PDQ-39, we conducted post-hoc analyses for the single index scores using ANCOVA in the same way as described above. To evaluate whether disease stage modified the intervention effect on our primary outcome, we conducted a post-hoc ANCOVA in the same way as described above. In this analysis, we studied the interaction effect between Hoehn & Yahr stage and group allocation. Lastly, we conducted a post-hoc sensitivity analysis (ANCOVA) using the stratification variables as covariates to verify robustness of the results.

### Role of funding

The funders were not involved in the writing process of the manuscript, nor involved in any other aspect pertinent to the study.

## Results

Between March 1, 2019, and March 27, 2021, 417 patients were screened for eligibility. Between March 3, 2020, and June 26, 2020, inclusion was paused because of the lockdown during the COVID-19 pandemic. Follow-up measurements took place until November 10, 2021. 214 patients were included, who were assigned randomly (1:1) to the intervention group (n = 109, 51%) or the control group (n = 105, 49%). For 136 participants, of whom 67.3% in the intervention group and 76.9% in the control group, assessors had no inkling in what group they were allocated. Their presumption was correct for 31.6% in the intervention group and 23.1% in the control group, and incorrect for 1 participant from the intervention group. This difference between the groups was not statistically significant (p = 0.195, Fisher's Exact test). For 23 participants, no presumption was filled in due to drop-out^10^ or human errors. For the 55 other participants, their presumption was incorrect for 2 participants, was correct for 32 in the intervention group and for 21 in the control group. Baseline characteristics were similar between both groups (Table 1). A total of 12 patients (6%) dropped out, of whom 10 dropped out between the T0 and T1 measurements, and two between the T1 and T2 measurements (Fig. 1).

**Table 1:** Demographics and baseline characteristics.

	Intervention (n = 109)	Control (n = 105)
**With participating caregiver**	64	62
**Age,** in years (mean; SD)	67.4 (8.5)	68.9 (8.5)
**Sex: women**	32 (29.4%)	26 (24,8%)
**Disease duration,** in years (mean; SD)	8.1 (5.8)	7.5 (6.0)
**Hoehn and Yahr stage** (n; %)		
1	25 (22.9)	29 (27.6)
2	59 (54.1)	39 (37.1)
3	13 (11.9)	22 (21.0)
4	9 (8.3)	12 (11.4)
5	3 (2.8)	3 (2.9)
**MDS-UPDRS**		
I (mean; SD)	10.2 (5.5)	10.9 (5.8)
II (mean; SD)	13.6 (6.7)	13.9 (6.6)
III (mean; SD)	28.2 (10.9)	28.5 (12.5)
IV (median; IQR)	1.0 (0.0–6.0)	0.0 (0.0–5.0)
**MMSE** (median, IQR)	29.0 (27.0–30.0)	29.0 (27.0–30.0)
**Word fluency** (mean; SD)	22.02 (6.61)	21.96 (6.45)
**Dysarthria severity** (n; %)		
Minimal dysarthria	18 (16.5)	27 (25.7)
Light dysarthria	58 (53.2)	48 (45.7)
Moderate dysarthria	27 (24.8)	24 (22.9)
Severe dysarthria	5 (4.6)	6 (5.7)
Very severe dysarthria	1 (0.9)	0 (0.0)

There were five serious adverse events (SAE) in the intervention group (5%) and five in the control group (5%), but these were judged not to be directly associated with the intervention or study procedures (verified by the medical ethical committee). During the study, the diagnosis changed from PD to atypical parkinsonism for one patient in the intervention group (1%) and four patients in the control group (4%). A total of twenty SLTs participated, of whom five quit during the study for various reasons that were entirely unrelated to the study protocol itself (retirement, or a change of professional focus or job).

In Table 2 and 3, for every outcome measure it is reported how many participants completed the assessment. Most missing data are a result of the COVID-19 pandemic, when assessments had to be conducted online instead of at the patients’ home. The 1:1 ratio between the intervention and control group stayed intact. Therefore, we did not impute the missing data.

**Table 2:** Primary and secondary outcomes—subjective measures.

Outcomes	T0		T1		T2		Estimated difference between intervention and control group at T1		Difference intervention group between T2 and T1	
							Mean (95% CI)	p value	Mean (95% CI)	p value
**PDQ-39** (score 0–100)^a^	n		n		n					
Intervention	109	28.5 (12.4)	104	24.7 (13.2)	99	26.6 (12.8)	−2.0 (−4.0 to 0.1)	0.056	2.4 (0.8–4.0)	**0.004**
Control group	105	26.9 (12.2)	100	25.5 (13.1)	NA	NA				
**ROMP (patient)**^a^										
Speech (score 7–35)										
Intervention	109	17.8 (3.6)	104	15.55 (4.2)	99	16.8 (4.4)	−1.3 (−2.2 to −5.2)	**0.001**	1.4 (0.7–2.1)	**0.000**
Control group	104	17.9 (4.4)	100	16.8 (5.0)	NA	NA				
Swallowing (score 7–35)										
Intervention	109	11.6 (3.5)	104	10.7 (3.6)	99	11.0 (3.9)	−0.58 (−1.2 to 0.1)	0.073	0.3 (−0.3 to 0.9)	0.271
Control group	104	11.1 (3.5)	100	10.9 (3.5)	NA	NA				
Drooling (score 9–45)										
Intervention	106	14.0 (9.0–18.0)	104	13.0 (9.0–18.0)	99	14.0 (9.0–18.0)	−0.22 (−1.1 to 0.7)	0.682	0.44 (−0.2 to 1.1)	0.200
Control group	105	13.0 (9.0–17.0)	100	13.0 (9.0–17.0)	NA	NA				
**VHI** (score 0–120)^a^^,^^b^										
Intervention	97	35.9 (16.2)	92	30.9 (17.5)	83	32.0 (19.1)	−4.9 (−8.0 to −1.8)	**0.002**	0.1 (−2.4 to 2.7)	0.912
Control group	93	35.6 (20.1)	86	34.4 (21.1)	NA	NA				
**EQ-5D** (score −0.33 to 1.0)^b^										
Intervention	99	0.78 (0.69–0.89)	93	0.81 (0.71–0.95)	85	0.78 (0.69–0.89)	0.01 (−0.27 to 0.05)	0.520	−0.02 (−0.05 to 0.02)	0.345
Control group	94	0.78 (0.69–0.86)	88	0.78 (0.68–0.89)	NA	NA				
**HADS** (score 0–21)^a^^,^^b^										
Intervention	98	8.0 (5.0–15.0)	93	7.0 (4.0–13.0)	84	7.5 (4.0–13.0)	−0.6 (−1.7 to 0.6)	0.333	−0.5 (−1.4 to 0.4)	0.292
Control group	94	8.5 (4.0–14.0)	88	8.0 (3.3–13.0)	NA	NA				
**ZBI-12** (score 0–48)^a^^,^^b^^,^^c^										
Intervention	59	7.0 (3.0–14.0)	57	7.0 (3.0–14.0)	49	9.0 (6.0–15.0)	−0.7 (−2.1 to 0.8)	0.354	1.8 (0.2 to 3.4)	**0.026**
Control group	61	7.0 (4.0–11.0)	59	7.0 (4.0–12.0)	NA	NA				
**ROMP -Speech (caregiver)** (score 7–35)^a^^,^^b^^,^^c^										
Intervention	57	16.7 (4.2)	56	15.3 (4.0)	49	15.8 (4.7)	−2.1 (−3.3 to −1.0)	**<0.001**	0.81 (0.00 to 1.6)	**0.040**
Control group	60	16.0 (3.7)	59	16.6 (4.8)	NA	NA				

Data are median (IQR), or mean (SD). Bold indicates the p values that are statistically significant.

Abbreviations: PDQ-39, Parkinson's Disease Questionnaire −39; ROMP, Radboud Oral Motor Inventory for Parkinson's Disease; VHI, Voice Handicap Index; EQ-5D, EuroQol-5 Dimension; HADS, Hospital Anxiety and Depression Scale; ZBI-12, Zarit Caregiver Burden Interview short form; NA, not applicable.

a Decrease in score over time suggests improvement.

b Surveys sent online. Despite sending reminders, not all participants filled in the online surveys, resulting in a lower n for marked outcomes.

c Surveys filled in by caregivers.

**Table 3:** Secondary outcomes–objective measures.

Outcomes	T0		T1		T2		Estimated difference between intervention and control group at T1		Difference intervention group between T2 and T1	
							Mean (95% CI)	p value	Mean (95% CI)	p value
**MRR/pa/**(syllables per second)										
Intervention	107	6.43 (1.02)	103	6.45 (1.02)	97	6.59 (1.01)	0.04 (−0.13 to 0.21)	0.650	0.09 (−0.11 to 0.30)	0.357
Control group	105	6.44 (1.11)	98	6.53 (0.94)	NA	NA				
**MRR/ta/**(syllables per second)										
Intervention	108	6.00 (1.22)	103	6.04 (1.11)	97	6.25 (1.01)	−0.04 (−0.24 to 0.16)	0.712	0.17 (0.00–0.35)	**0.047**
Control group	105	6.06 (1.01)	98	6.18 (1.00)	NA	NA				
**MRR/ka/**(syllables per second)										
Intervention	107	5.38 (1.10)	102	5.54 (1.02)	96	5.56 (1.09)	0.11 (−0.07 to 0.28)	0.222	0.05 (0.08–0.18)	0.481
Control group	104	5.49 (1.08)	98	5.61 (1.06)	NA	NA				
**MRR/pataka/**(syllables per second)										
Intervention	108	6.15 (1.06)	103	6.22 (1.19)	97	6.27 (1.30)	0.07 (−0.14 to 0.27)	0.512	0.06 (−0.16 to 0.28)	0.596
Control group	105	5.99 (1.24)	97	6.04 (1.19)	NA	NA				
**MPT** (seconds)										
Intervention	108	18.53 (13.96–24.87)	103	18.00 (14.10–23.93)	98	17.91 (13.85–23.50)	−0.25 (−1.68 to 1.18)	0.731	0.24 (−1.05 to 1.52)	0.714
Control group	105	15.65 (11.98–22.17)	98	16.30 (12.74–23.55)	NA	NA				
**MPR** (semitones)										
Intervention	109	24.2 (6.2)	103	24.4 (6.9)	98	25.7 (6.3)	−4.0 (−1.8 to 1.0)	0.580	1.2 (0.1–2.3)	**0.031**
Control group	105	24.5 (6.6)	98	24.5 (6.9)	NA	NA				
**MLL** (dB)^b^										
Intervention	108	103.0 (9.2)	91	104.3 (8.2)	82	104.3 (8.9)	−1.1 (−2.4 to 0.2)	0.089	1.3 (0.1–2.4)	**0.029**
Control group	105	102.3 (9.0)	85	103.8 (7.8)	NA	NA				
**AVQI** (score 0–10)^a^^,^^b^										
Intervention	108	4.69 (1.28)	90	4.29 (1.15)	81	4.32 (1.28)	−1.00 (−0.40 to 0.21)	0.536	0.13 (−0.18 to 0.43)	0.406
Control group	105	4.84 (1.50)	85	4.53 (1.25)	NA	NA				
**MSS** (timed test) (ml/s)^b^										
Intervention	105	13.9 (7.0)	91	14.9 (8.3)	81	15.1 (8.1)	0.6 (−0.6 to 1.8)	0.318	−0.6 (−1.7 to 0.5)	0.281
Control group	102	13.0 (7.1)	85	12.47 (6.27)	NA	NA				
**DIT** (percentage)^c^										
Intervention	108	98.3 (96.6–99.2)	99	98.4 (97.4–100)	96	98.3 (96.8–100)	2.1 (0.3–3.9)	**0**.**024**	−0.4 (−1.1 to 0.4)	0.320
Control group	104	98.3 (95.0–99.8)	96	98.3 (94.4–99.2)	NA	NA				

Data are median (IQR), or mean (SD). Bold indicates the p values that are statistically significant.

Abbreviations: PDQ-39, Parkinson's Disease Questionnaire −39; MRR, maximum repetition rate; MPT, maximum phonation time; MPR, maximum pitch range; MLL, maximum loudness level; AVQI, acoustic voice quality index; MSS, maximum swallowing speed; DIT, Dutch intelligibility test (sentence level); NA, not applicable.

a Decrease in score over time suggests improvement.

b During the COVID-19 pandemic, a part of the T1 and T2 assessments had to be conducted online. Not all assessment could be conducted remotely, resulting in a lower n for marked outcomes.

c The percentage scores at baseline almost reached the maximum of 100% for all patients, making further improvement impossible.

### Primary outcomes

**Fig. 2:**
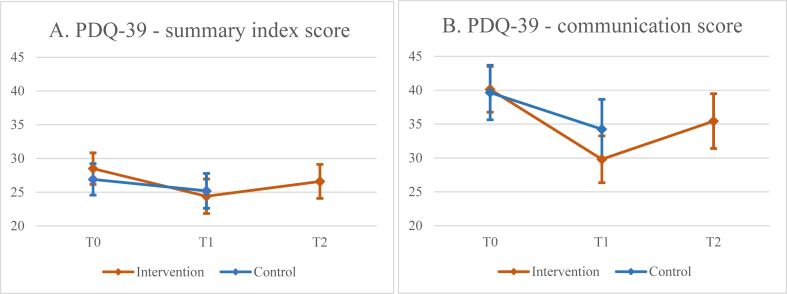
**A.** Primary analysis: the observed summary index score of the Parkinson’s Disease Questionnaire 39 (PDQ-39) for the intervention and the control group (score 0-100). 95% confidence intervals are displayed. **B**. Post hoc analysis: the observed communication index score of the PDQ-39 for the intervention and the control group (score 0-100). 95% confidence intervals are displayed.

At the primary endpoint of 8 weeks, the intervention group did not have a significantly improved health-related quality of life (summary index score of the PDQ-39) compared to the control group (p = 0.056; Fig. 2a, Table 2). The estimated mean difference in PDQ-39 between the intervention group and the control group was −2.0 (95% CI −4.0 to 0.1), in favour of the intervention group. A post-hoc sensitivity analysis using stratification variables as covariates showed no notably differences in the summary index score of the PDQ-39 with an estimated difference of −2.0 (95% CI −4.0 to 0.4), p = 0.054, demonstrating the robustness of our findings. Post-hoc analyses showed that out of the total of the eight single index scores of the PDQ-39, only the communication index score showed an improvement (p = 0.011) in favour of the intervention group, with an estimated mean difference of −5.3 (95% CI −9.4 to −1.2) (see Appendix). Furthermore, post-hoc analyses showed no interaction effect of Hoehn & Yahr stage and group allocation on health-related quality of life (interaction effect = −652, CI = −1.36 to 2.67, p-value = 0.524.), suggesting that disease stage did not affect the results much.

### Secondary outcomes

For the secondary outcomes, significant benefits in favour of the intervention group were found for voice-related complaints (VHI) and severity of speech complaints as reported independently by both the patient (ROMP) and the caregiver (ROMP adapted to caregiver), see Table 2. There were no notably differences in the other secondary outcomes between both groups.

### Long-term follow-up measurement (32 weeks)

At the secondary endpoint (T2), we found a significant reduction in disease-related quality of life on summary index score of the PDQ-39 compared to T1, reflected in a higher score. A significant decline was also found for the ROMP Speech and for the ROMP adapted to the caregiver. For the objective speech-related outcomes, a significant improvement was found for maximum loudness level, maximum pitch range, and the maximum repetition rate of the syllable/ta/. Finally, a decline in caregiver burden was found with the Zarit caregiver Burden Interview Short form. There were no other significant differences at the secondary endpoint. An overview of all secondary outcomes can be found in Table 2 and 3.

## Discussion

We found that remotely delivered and personalised speech therapy (the PERSPECTIVE intervention) did not significantly improve disease-related quality of life in general. Immediately following treatment, quality of life was better in the treatment arm, with a difference that exceeded the hypothesised minimally clinically important difference of 1.6 points on the PDQ-39,^33^ but this difference did not reach statistical significance. Although the study was not powered for post-hoc analyses, secondary analyses did suggest that remote speech therapy improved the communication sub-score of self-reported quality of life, but not any of the subdomains of the PDQ-39, suggesting a genuine effect driven by improvements in speech. Additional secondary analyses also showed an improvement in the self-reported severity of voice and speech complaints, as reported independently by both the patients and caregivers. Objectively measured speech outcomes did not differ significantly between both study arms. Of the observed improvements after speech therapy, only the VHI-score was retained at 6 months follow-up.

We opted for disease-related quality of life as an ambitious primary treatment goal, since we believe that the ultimate goal of any treatment for persons with PD should be to improve their quality of life. Our motivation to include quality of life as primary outcome came from the notion that speech is an important factor contributing to quality of life for persons with PD. At the same time, we do acknowledge that quality of life is a challenging outcome that is determined not just by speech but also by a wide variety of other domains, many of which are not targeted specifically by speech therapy. We also acknowledge that quality of life measurement tools such as the PDQ-39 lack discriminative validity, certainly when applied in a heterogenous PD population (as we did) where patient performance varies considerably over time, often for reasons unrelated to the study intervention. In that regard, measures of change in speech are arguably better outcomes as these offer proof-of-concept evidence for target engagement. Post-hoc analyses showed that the trend towards an improved general quality of life in the intervention group was driven by only one of the eight domains, namely the communication-related quality of life, which is in line with a genuine treatment effect. This improvement in the communication subdomain exceeded the minimally important difference of 4.2 for this domain. Although this is only a small part of the overall PDQ-39, being able to communicate independently is an important aspect of quality of life. The absence of change in other domains of the PDQ-39 after treatment actually, supports our hypothesis that speech therapy specifically improves communication and thereby quality of life.

Our findings are largely comparable to two smaller earlier studies. A noninferiority study comparing online speech therapy to face-to-face therapy found a significant improvement in the communication subdomain for PD patients who received online speech therapy, but no improvement in overall quality of life.^13^ Another RCT of speech therapy in PD also found an improvement in speech outcomes and communication effectiveness.^8^ Contrary to our trial, this latter study also noted an improvement in objective speech measurements. However, in both other studies, the intervention was delivered at high intensity for all participants, whereas we personalised the treatment according to what participants could tolerate. Moreover, the objective speech measures used in this study^8^ (sound pressure level during reading tasks and monologue) are hard to compare with the objective outcome measures in this trial (maximum performance tasks). This difference might explain the improvments of the objective speech measures in the study by Ramig and colleagues.^8^ An important advantage of the PERSPECTIVE study compared to other studies is the inclusion of participants with a broad range of disease severity, making this a more real-life intervention with greater external validity. Specifically, although we included overall more people in early disease stages, we also recruited 62 participants in Hoehn & Yahr stage III, IV and V, giving valuable information on this relatively underrepresented group in PD research. This broad inclusion was enabled by the remote delivery of the treatment, and also by the personalised nature of the intervention which was tailored to the abilities and treatment goals of each individual.

Based on earlier results of Ramig and colleagues, who found a retained improvement of a participant-reported Modified Communication Effectiveness Index (CETI-M) 6 months after speech therapy,^8^ we expected that the observed improvements of the self-rated secondary outcomes in our trial would also persist at long-term follow-up. However, this was only the case for the VHI and not for the ROMP speech. To enhance the validity of our findings, we included a real-life sample of patients with any degree of speech complaints, also including those with modest treatment goals and those who could not comply with highly intensive therapy because of less endurance, more cognitive problems, or greater dependency on caregivers. Although intensive treatment is considered as the optimal approach for persons with PD to regain speech intelligibility, realistically however, such an intensive treatment is not feasible for all patients. This could explain why in this trial, not all observed effects were retained at follow-up. As such, the results of this trial offer a realistic view on actual daily clinical practice, suggesting that some form of maintenance therapy is likely required How to achieve such a retainment deserves further study.

An important aspect of our intervention was the fully remote delivery in the patient's own homes using telerehabilitation, making this treatment theoretically accessible to many people with PD around the world, provided they have a functioning internet connection. Occasional problems were reported with maintaining a stable internet connection, but telerehabilitation worked well for most participants. We appreciate that the experience may be less gratifying in other parts of the world where IT networks are less well developed. But overall, our findings do suggest that telerehabilitation offers an opportunity to make specialised and high-quality speech therapy accessible to more persons with PD, since the distance to a therapist is no longer a limitation. This may ultimately also improve the quality of care, because therapists will be able to increase their caseload and hence become progressively more experienced to treat a specific group of patients, while more patients are able to choose to be treated by a specialised therapist. Our experience in The Netherlands is that specialised treatment by allied health therapists treating a high case load is associated with better patient outcomes and lower cost.^34^^,^^35^ The remote element of our intervention will also be helpful in delivering a low intensity maintenance therapy to achieve a more long-term persistence of the therapeutic benefits.

We included two swallowing outcomes because pilot studies^36^^,^^37^ suggested that LSVT in PD may also improve swallowing and cough effectiveness, via activation of the laryngeal system. However, we found no difference in swallowing capacity, as measured by maximum swallowing speed, or swallowing complaints as measured by a validated PD specific questionnaire. One possible explanation is the relatively low severity of swallowing complaints at baseline, conceivably because we purposely included PD patients with speech complaints, but not necessarily those with dysphagia. Another explanation is that there is a large discrepancy between subjective prevalence of dysphagia (about one third) and objective prevalence of dysphagia in PD (57–80%)^38^^,^^39^ and therefore, our measurements are less sensitive to finding dysphagia. Whether intensive speech treatment may ameliorate swallowing remains to be studied in a large dedicated study combining a range of subjective and objective measures, performed in participants with clinically relevant dysphagia.

Several limitations of this study should be mentioned. First, our outcome measures presented several challenges. Besides the already mentioned lack of discriminative validity, quality of life as a primary outcome is also potentially affected by several confounding variables, and we were not able to measure all of them (e.g. medication status, sleep, or cognitive challenges). However, since participants were randomised at baseline, we assume that both study groups were comparable to each other, also for factors that we did not measure. Some outcome measures of speech may not have provided a realistic view of daily functioning, since they were assessed in a test situation. This applies in particular to the assessments of speech intelligibility and the maximum performance task. When participants know that their speech is being evaluated, the assessment itself may act as an incentive to speak more intelligibly or to perform better than without being supervised.^40^ Moreover, in daily life patients must rely more on cognitive functions, e.g. while maintaining a conversation with background noise. We did not assess speech intelligibility with these influencing factors. Future research could use more advanced techniques like intelligibility in the presence of background noise such as babbling^41^ to create a more realistic communication environment. Moreover, maximum performance tasks and speech intelligibility are widely used and helpful dysarthria assessments,^42^ but may not be sufficiently sensitive in detecting changes in hypokinetic speech.

Second, since we compared the intervention group to a waiting list instead of a sham intervention, it is possible that the ‘tender, loving care’ effect could have influenced our results in a positive way.^43^ However, out of all the measurements that we conducted, only the speech related outcomes improved, suggesting that the intervention only showed an effect specifically on speech instead of on overall wellbeing. This effect is particularly visible in the outcomes of the subscales of the PDQ-39, where only the communication subscale improved. Also, anxiety and depression status as measured by the HADS did not differ between and within study groups. This makes it less likely that a more global tender loving care effect influenced our results.

Third, a part of the data collection was conducted during the COVID-19 pandemic. Since the intervention was delivered via telemedicine, the pandemic did not hamper the continuation of online speech therapy, which is a strength. However, not all assessments could be completed since a part of them had to be conducted online instead of at the patients’ home, resulting in missing data. Although we cannot completely rule out that the pandemic influenced our results, the circumstances regarding the COVID-19 pandemic did not differ for the intervention and control group as a result of the randomisation. Therefore, we expect that possible effects of the pandemic have the same size between both groups and no data were imputed. Furthermore, the safety measures did impact the assessment visits. Baseline home visits were delayed until the lockdown restrictions were lifted. To continue the study, part of the planned T1 and T2 visits had to be conducted online. This made it impossible to conduct some of the assessments, such as maximum loudness level, swallowing speed and the physical examination of the MDS-UPDRS. However, the latter was used only for baseline descriptive purposes, and these measurements were complete.

Furthermore, while participants were instructed not to disclose their group allocation, some participants accidentally revealed it to the assessor, thereby de-blinding the assessor. In addition, some participants spoke remarkably better at the follow-up assessment based on which the assessor might have guessed group allocation. There is a possibility that this influenced some of the results. However, the primary outcome (PDQ-39) is a questionnaire completed by the participant, hence this was not affected by the assessor's presumption. The presumptions may have had an effect on the outcomes that are sensitive to the assessor's influence (such as the maximum performance speech tasks), but we did not find any significant differences for these outcomes between the intervention and the control group, which would in fact suggest that the influence of the assessor was negligible.

Lastly, as in most other studies in PD,^44^ a relatively low proportion of women participated in our study (27%, which is less than the proportion of women in the Dutch PD population, estimated to be 42%^45^). Other studies of speech therapy in PD had a similar recruitment issue.^5^ We have no specific reason to believe that the remote speech therapy benefited men and women differently, yet every effort must be made to make speech therapy accessible to everyone with PD, both in research and daily care. More work is also needed to better understand how speech therapy should be optimally personalised for people with PD.

## Contributors

B.R. Bloem, N.M. de Vries, and J.G. Kalf conceived the study and initiated the study design. J.J.L. Maas performed the study and analysed the results together with J. IntHout. J.J.L. Maas accessed and verified the data. All authors contributed to the final manuscript.

## Data sharing statement

We will make aggregated and anonymised data available in a validated database. Access to the data is restricted, meaning that researchers who are interested in re-use of the data are asked to contact the central contact person for permission. Approval is given after a signed agreement. Data will be available beginning 1 year after publication.

## Declaration of interests

J.J.L. Maas has nothing to declare. N.M. de Vries reports grants from the Netherlands Organisation for Health Research and Development (ZonMw), Michael J Fox Foundation, and Verily Life Sciences, has received a travel stipend to visit the World Parkinson Congress, serves in the advisory board of the Royal Dutch Society for Physical Therapy, and serves as the associate editor of the Journal of Parkinson's disease. J. IntHout has nothing to declare. B.R. Bloem serves as the co-Editor in Chief for the Journal of Parkinson's disease, serves on the editorial board of Practical Neurology and Digital Biomarkers, serves on the executive scientific advisory board of the Michael J fox Foundation for Parkinson Research, serves as officer (Secretary) of the Movement Disorder Society, has received fees from serving on the scientific advisory board for the Critical Path Institute, Gyenno Science, MedRhythms, UCB, Kyowa Kirin and Zambon (paid to the Institute), has received fees for speaking at conferences from AbbVie, Bial, Biogen, GE Healthcare, Oruen, Roche, UCB and Zambon (paid to the institution), and has received research support from Biogen, Cure Parkinson's, Davis Phinney Foundation, Edmond J. Safra Foundation, Fred Foundation, Gatsby Foundation, Hersenstichting Nederland, Horizon 2020, IRLAB Therapeutics, Maag Lever Darm Stichting, Michael J Fox Foundation, Ministry of Agriculture, Ministry of Economic Affairs & Climate Policy, Ministry of Health, Welfare and Sport, Netherlands Organization for Scientific Research (ZonMw), Nothing Impossible, Parkinson Vereniging, Parkinson's Foundation, Parkinson's UK, Stichting Alkemade-Keuls, Stichting Parkinson NL, Stichting Woelse Waard, Health ∼ Holland/Topsector Life Sciences and Health, UCB, Verily Life Sciences, Roche and Zambon. J.G. Kalf has received grants from the Michael J Fox Foundation, Michel Keijzerfonds, Ataxie Vereniging Nederland, and from the Netherlands Organisation for Health Research and Development (ZonMw) (all paid to the institution), has received a fee for lecturing for the International Parkinson & Movement Disorders Society, has received a travel stipend to visit the World Parkinson Congress, and has served as councillor of the board for the Dysphagia Research Society (2021–2022).

## Appendix A Supplementary data

Supplementary Table

Study protocol
