# Supplementary material for: Effectiveness of remotely delivered speech therapy in persons with Parkinson's disease – a randomised controlled trial
Source: eClinicalMedicine. 2024 Sep 11;76:102823. doi: 10.1016/j.eclinm.2024.102823 (PMC11415969; doi:10.1016/j.eclinm.2024.102823)
Supplement: Supplementary Figure [file mmc1.docx]

**Interface of the Voice trainer**

| 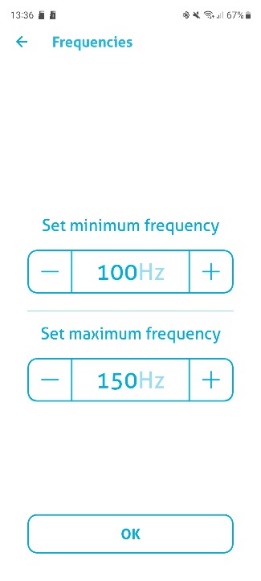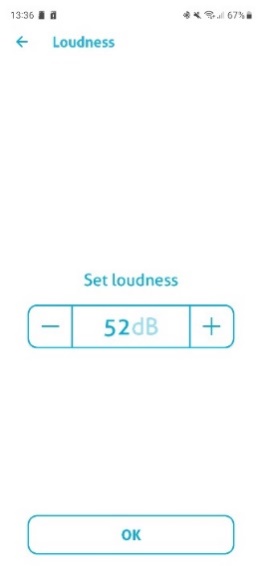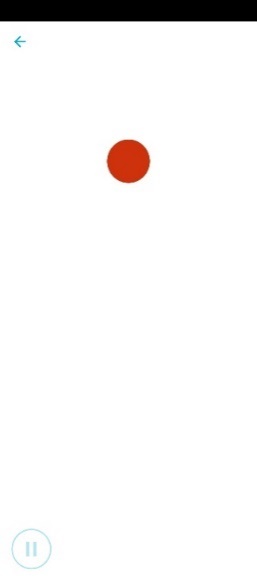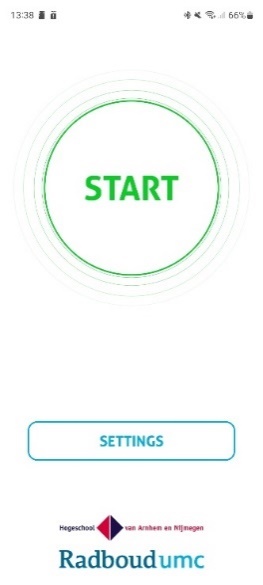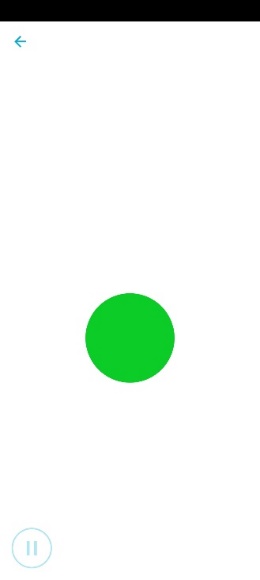 | | | | |
| --- | --- | --- | --- | --- |
| **A** | **B** | **C** | **D** | **E** |

**Figure 1.** Screenshots of the Voice trainer**. A.** home screen of the Voice trainer **B.** setting the loudness level (dB) **C.** setting the frequency range (Hz) **D/E.** visual feedback about pitch and loudness level using a circle.

**Explanation**The Voice trainer provides real-time visual feedback on pitch and loudness of speech, particularly helpful for people with hypokinetic dysarthria. If the circle is green and large, this means the speech is loud enough, i.e. louder than the pre-set dB level. If the circle becomes red and small, the user speaks too softly. The placement of the circle reflects pitch: when the circle moves upward in the screen, the pitch is too high. Ideally, the circle is placed in the middle or slightly under the middle of the screen to support intelligible speech.
