# Supplementary material for: Effectiveness of remotely delivered speech therapy in persons with Parkinson's disease – a randomised controlled trial
Source: eClinicalMedicine. 2024 Sep 11;76:102823. doi: 10.1016/j.eclinm.2024.102823 (PMC11415969; doi:10.1016/j.eclinm.2024.102823)
Supplement: Supplementary Table [file mmc2.docx]

**Appendix**

| **Parkinson’s Disease Questionnaire 39 domain** | **T1 index scores**  **intervention group** | **T1 index scores**  **control group** | **Estimated difference between intervention and control group at T1,**  **mean (95% CI)** | **P value** |
| --- | --- | --- | --- | --- |
| Mobility | 25.3 | 26.3 | -1.0 (-4.1 to 2.1) | 0.518 |
| Activities of daily living | 30.7 | 33.7 | -3.0 (-6.2 to 0.2) | 0.067 |
| Emotional wellbeing | 22.4 | 23.1 | -0.8 (-3.8 to 2.2) | 0.617 |
| Stigma | 13.9 | 13.6 | 0.33 (-3.1 to 3.8) | 0.850 |
| Social support | 15.0 | 18.4 | 3.4 (-7.2 to 0.47) | 0.085 |
| Cognition | 28.5 | 27.5 | 1.08 (-1.9 to 4.1) | 0.478 |
| Communication | 29.4 | 34.7 | -5.3 (-9.4 to -1.2) | **0.011** |
| Bodily discomfort | 30.9 | 34.4 | -3.5 (-7.9 to 1.0) | 0.124 |
| Total score | 23.8 | 25.9 | -2·0 (-4·0 to 0·1) | 0.056 |

Table 1. Post hoc analyses: estimated mean index scores of the Parkinson’s Disease Questionnaire 39. Outcomes are not adjusted for multiple testing.
