# Supplementary material for: Effectiveness of remotely delivered speech therapy in persons with Parkinson's disease – a randomised controlled trial
Source: eClinicalMedicine. 2024 Sep 11;76:102823. doi: 10.1016/j.eclinm.2024.102823 (PMC11415969; doi:10.1016/j.eclinm.2024.102823)
Supplement: Study protocol [file mmc3.pdf]

Maas et al. Trials (2022) 23:274
https://doi.org/10.1186/s13063-022-06160-9

Trials

STUDY PROTOCOL Open Access

Design of the PERSPECTIVE study: PERsonalized SPEeCh Therapy for actIVE conversation in Parkinson's disease (randomized controlled trial)

Check for updates

J. J. L. Maas[1*], N. M. De Vries[1], B. R. Bloem[1] and J. G. Kalf[2]

Abstract

Objective: To evaluate the effectiveness of personalized and home-based speech therapy on quality of life, intelligibility, and social participation for people with Parkinson's disease (PD) who have a reduced intelligibility of speech.

Background: Speech problems in PD have a profound negative impact on social interaction and quality of life. Evidence for speech therapy in PD is growing, but more work remains needed to explore its full potential. Efficacy exists for highly intensive standardized speech treatment programs, but not all patients can comply with this rather intense intervention, especially the more severely affected ones. Here, we aim to study the effectiveness of personalized and home-based (remote) speech therapy in PD on quality of life and speech. The intervention will be supported by a dedicated speech training app. We expect that this approach will improve speech intelligibility and quality of life in patients irrespective of disease stage.

Methods: We will perform a single blind, randomized controlled trial, comparing 8 weeks of speech therapy to no intervention using a waiting list design. A total of 215 PD patients with problems in intelligibility will be recruited by 12 highly experienced speech therapists. All patients will be measured at baseline and after 8 weeks (primary endpoint). Additionally, the experimental group will be re-assessed one more time, after a wash-out period of 24 weeks. The control group will receive deferred treatment after 8 weeks, but without additional follow-up assessments. Our primary outcome is quality of life (as measured with PDQ-39). Secondary outcomes include speech and voice quality, intelligibility, severity of voice and speech complaints, and caregiver burden.

Results: The inclusion of participants has started on March 1, 2019, and is expected to be finalized on April 1, 2021. We expect to have the first results in January 2022.

* Correspondence: janna.maas@radboudumc.nl
[1]Donders Institute for Brain, Cognition and Behaviour, Department of Neurology, Centre of Expertise for Parkinson & Movement Disorders, Radboud University Medical Centre, Nijmegen, The Netherlands
Full list of author information is available at the end of the article

© The Author(s). 2022 **Open Access** This article is licensed under a Creative Commons Attribution 4.0 International License, which permits use, sharing, adaptation, distribution and reproduction in any medium or format, as long as you give appropriate credit to the original author(s) and the source, provide a link to the Creative Commons licence, and indicate if changes were made. The images or other third party material in this article are included in the article's Creative Commons licence, unless indicated otherwise in a credit line to the material. If material is not included in the article's Creative Commons licence and your intended use is not permitted by statutory regulation or exceeds the permitted use, you will need to obtain permission directly from the copyright holder. To view a copy of this licence, visit http://creativecommons.org/licenses/by/4.0/. The Creative Commons Public Domain Dedication waiver (http://creativecommons.org/publicdomain/zero/1.0/) applies to the data made available in this article, unless otherwise stated in a credit line to the data.

Conclusions: We will investigate the effectiveness of speech therapy in PD. Particular strengths of our study include a randomized and single-blinded design, the personalized treatment approach, the inclusion of PD patients irrespective of disease stage or severity of the speech complaint, the long-term follow-up, the adequate power, and the use of a patient-relevant primary endpoint. This will allow us to draw firm conclusions about the effectiveness of personalized and remote speech therapy for PD patients in all disease stages.

Trial registration: ClinicalTrials.gov NCT03963388. Registered on May 24, 2019

Keywords: Parkinson's disease, Speech therapy, Telemedicine, Randomized controlled trial

Background

Parkinson's disease (PD) is a progressive neurological disorder, with a large variety of motor and non-motor problems, including reduced speech intelligibility which may occur in up to 70% of patients [1]. Even patients without explicit intelligibility problems can have difficulties conducting a routine conversation. Reduced intelligibility and poor communication skills can have a profound negative impact on social interaction and quality of life [2]. Since pharmacological treatment only has limited beneficial effects on speech [3, 4], speech therapy is particularly relevant to improve speech quality and to enhance intelligibility.

Currently, there is level II evidence to support existing speech treatment programs in PD [5, 6]. The most recent Cochrane reviews from 2012 on this subject showed that evidence is growing, but more work remains to explore its full potential due to small sample sizes, inadequate methodology, lack of outcomes relevant to PD patients, and insufficient follow-up to determine the duration of any improvement [6, 7]. Since 2012, three further RCTs have been initiated [8–10], all aiming to evaluate Lee Silverman Voice Treatment LOUD (LSVT-LOUD) versus another or no treatment in a small sample (20 and 30 patients per treatment arm).

It seems that current speech treatments that show positive outcomes are not suitable for all PD patients: LSVT-LOUD [10–12], Pitch Limiting Voice Treatment [13], and Speak Out [14] are all highly intensive (3–4 times a week in a professional setting, plus home exercises) and generally follow the same program for all patients. A recent LSVT analysis reported that, in routine care, some patients refuse this high-intensity treatment or do not adhere to the entire protocol because of physical limitations or fatigue, suggesting that this intensive approach is most suitable for only a select group of patients [15]. Offering treatment remotely (i.e., delivered at home) may be an attractive solution to increase treatment intensity, while reducing travel burden. Recently, studies using telerehabilitation have shown the feasibility of online delivery of speech therapy in small samples of PD patients [16, 17], but dedicated speech therapy is still not available for many PD patients, in particular, for those with advanced PD.

In addition to the research challenges mentioned above, it is also common to experience various unmet challenges related to speech therapy in PD itself. First, although it is relatively easy to temporarily increase intelligibility in PD patients with the right instruction, it takes intensive exercise and specific feedback for patients to get used to normal loudness again and to apply it independently in an everyday conversation. A second challenge is that speech exercise can make speech more intelligible, but patients may remain dependent on cueing. It is uncertain how to deliver such cueing at home, outside the therapeutic settings. Thirdly, conveying a message through speech requires producing both comprehensible language and intelligible speech at the same time. This dual tasking becomes increasingly difficult for patients with PD [18], making them progressively more dependent on caregivers. To help patients to monitor their speech, we have developed a sophisticated app, namely the Voice trainer [19], that provides real-time visual feedback aiming to improve intelligibility. Several user tests were conducted with both patients and speech therapists to optimize the usability of the application and before introducing and implementing the app in the training of speech therapists in the Netherlands. In this study, patients use the voice trainer to practice their speech quality and maintain their intelligibility during daily conversation and social interaction as well.

In an experimental design, our main aim is to address all these challenges by evaluating a multifaceted approach of home-based speech therapy in a single-blind, randomized, and controlled trial, using quality of life as the primary outcome. Specifically, our approach combines the following elements: (1) personalized treatment, based on (internationally) established speech treatment programs and guidelines [20, 21], making speech therapy available for every PD patient regardless of disease stage; (2) consistent use of the Voice Trainer app to enable cueing during treatment and outside the therapeutic setting; and (3) treatment delivery within the patients' home using telerehabilitation and delivered by highly experienced therapists who work as part of the Dutch ParkinsonNet (a professional network of allied health therapists with dedicated expertise in the management

of PD [22, 23]), to enable patients to follow the program with adequate intensity at home.

We expect an improvement in both quality of life (primary outcome) and speech quality (secondary outcome) for patients across all disease stages. Moreover, we hypothesize that improvements will be maintained at follow-up, since long-term effects in improvements of speech in PD have been found in previous studies [10]. Therefore, our primary objectives are (1) to study the effectiveness of personalized speech therapy on quality of life in patients with Parkinson's disease and (2) to explore whether the effects of speech therapy remain after long-term follow-up (6 months).

Methods/design

Trial design

Here, we will perform a single-blinded randomized controlled trial comparing speech therapy to no intervention using a waiting list design. We opted for this design because this enables us to properly compare patients who have received therapy with patients who have not received therapy (yet), thereby answering our primary research question addressing the effectiveness of speech therapy in patients with PD. The control group receives deferred treatment directly after reassessment, which is a realistic waiting period in usual care. Patients will be assigned to either the experimental group or the control group in a 1:1 manner, by means of a computerized validated variable block randomization model using Castor EDC [24] with automatically generated block sizes of 4, 6, and 8. Stratification will take place for gender, speech therapist, Hoehn and Yahr stage, and age (< 46 years, 47–55 years, 56–65 years, > 66 years). To ensure blinding, allocation of the participants is made invisible for assessors in the data collection program Castor EDC. Furthermore, patients and speech therapists are instructed to refrain from discussing the treatment with the assessors. Measurements will be performed at baseline (T0) and after 8 weeks (T1). For the intervention group only, an additional follow-up measurement takes place 32 weeks after baseline (T2) (Fig. 1) to study whether the effects are retained at the long term. The T1 measurement is the primary endpoint at which we will compare the intervention group with the control group. The T2 measurement is the secondary endpoint at which we will compare the within-group differences of the intervention group after follow-up.

Full ethical approval has been granted by the medical ethical committee of Arnhem-Nijmegen (NL67867.091.18), and the PERSPECTIVE trial is registered at ClinicalTrials.gov (NCT03963388) on May 24, 2019. Important protocol modifications will receive a written notice to the relevant parties (medical ethical committee, trial register, speech therapists, and/or participants).

Procedure

Patients are asked to participate by highly experienced speech therapists, and we will recruit these speech therapists throughout the Netherlands, using the nationwide ParkinsonNet infrastructure [23]. ParkinsonNet is a national health care model that consists of 70 regional networks, with over 3000 health professionals with specific expertise in the treatment of PD patients [22]. Twelve speech therapists who participate in ParkinsonNet will be selected based on their caseload, who will each treat 16–20 patients, which is approximately 1 patient per month during the inclusion period.

During the first visit, speech therapists inform eligible patients about the study with a formal information letter. The speech therapists explicitly do not give any therapeutic advice to patients during this first visit, since patients can already benefit from education. To provide the patients enough time to consider participation, the research team calls the patients after 7 days to verify their willingness to participate and to check for eligibility. When a participant is willing and eligible, the baseline measurement (T0) is scheduled. One day after baseline measurement, randomization is performed by a researcher who is not involved in any of the measurements and who also subsequently informs both the therapist and patient about the group allocation. If the patient is allocated to the intervention group, treatment will start within a week. If the patient is allocated to the control group, treatment will start after the second measurement (T1), as shown in the flowchart (Fig. 1).

The intervention is delivered during a maximum period of 8 weeks. The number of sessions depends on the complexity of goals and possibilities of the patient (i.e., time and availability). Between T1 and T2 (only for the intervention group), patients will not receive speech therapy but still can use the Voice Trainer app.

Participants

Eligible patients have PD according to the MDS criteria [25] as confirmed by their own treating neurologists. Other inclusion criteria are (1) reduced intelligibility bothering daily communication according to the patient and/or informal caregiver(s), (2) a desire to improve their speech, and (3) willingness and ability to receive online treatment. Importantly, patients who do not own a device that is suitable for online treatment can still participate in the study, because in those cases, we will provide them with a loan device. The exclusion criteria are (1) recently received (< 1 year) speech therapy, (2) voice or speech problems due to other causes, (3)

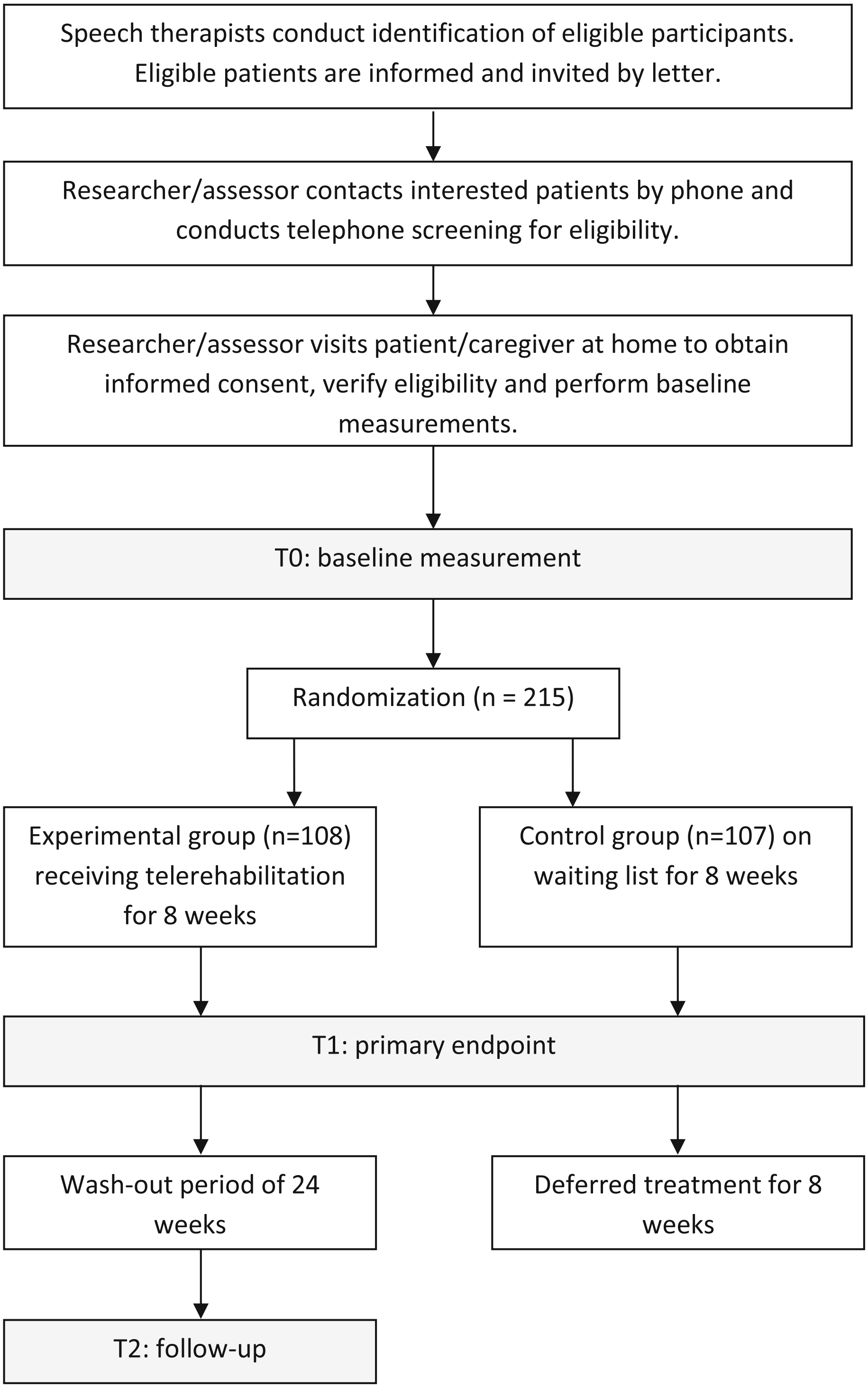

Fig. 1 Flowchart of the design and enrollment procedures

communication difficulties based on language problems without predominantly reduced intelligibility, and (4) (technical) inability to receive online treatment. Patients are not excluded by disease stage or the severity of their speech complaint, because we aim to study the effectiveness of the intervention irrespective of dysarthria severity.

The drop-out criteria are (1) withdrawal from consent and (2) health problems of such severity that conducting assessments is not possible. When willing and available,

a primary informal caregiver of each patient can participate in the study by completing two questionnaires about speech and caregiver burden. Inclusion will take place over a period of 25 months, starting on March 1, 2019, and ending on March 31, 2021. Informed consent of both patient and caregiver (when applicable) is obtained by the researcher before the baseline assessment.

Intervention

The PERSPECTIVE treatment approach combines three elements: (1) personalized treatment, (2) consistent use of a feedback app for smartphone or tablet that delivers real-time visual feedback (the Voice Trainer app), and (3) treatment delivery in the patient's home using telerehabilitation.

Personalized treatment

Based on the Guidelines for Speech-Language Therapy in Parkinson's Disease [26] and Pitch Limiting Voice treatment protocol [20], the intervention consists of 12–16 sessions (30–60 min each) in up to 8 weeks, with a maximum of three times a week and daily home exercise. The first treatment session includes an extensive assessment of the patient's personal conversational goal [27, 28], taking into account personal circumstances and (social) environment. The caregiver is also explicitly involved and, when necessary, instructed on how to help or support the patient in doing exercises or providing trained cues. The technique to improve intelligibility will be personalized, based on the patient's individual treatment goal. This treatment paradigm makes the treatment suitable for patients with mild to advanced hypokinetic dysarthria and has been successfully implemented in the Dutch ParkinsonNet for over a decade.

Support by feedback app

People with PD are highly dependent on feedback to improve motor control [29]. We therefore developed a dedicated app that provides real-time visual feedback about speech loudness and pitch: the Voice Trainer app [19]. The app supports patients and therapists to correctly practice and maintain voice loudness and pitch, during exercises as well as during conversation. As such, it supports patients in self-management and treatment compliance beyond the therapy sessions. The feasibility of the app has been confirmed by patients, caregivers, and speech therapists [30], and the use of the app has been integrated into the recently updated Guidelines for Speech-Language Therapy in Parkinson's Disease [31].

Highly experienced therapists who deliver online treatment

The intervention will be delivered by highly experienced speech-language therapists who participate in the Dutch national ParkinsonNet, who have all received a dedicated 3-day training program in treating PD patients according to evidence-based guidelines, and who all treat a high caseload in their daily practice which further increases their treatment quality [22, 23]. Delivery of the online treatment will be done using a reliable, certified, and secured online platform, which is easy to use without extra costs for patients (software provided by Zaurus B.V.). Speech therapy itself will be reimbursed by the Dutch healthcare insurance, but every participating therapist is offered a small fee of €50 per patient to compensate for the extra time they spend on training and coaching (see next paragraph), thereby facilitating them to participate in the study.

Training and coaching of trial therapists

Before the start of the trial, participating speech therapists follow a 2-day training in which they are informed about the study procedures and the PERSPECTIVE intervention protocol, including delivering online treatment. During the study period, speech therapists will be coached online by one of two experts to ensure that every patient receives optimal speech therapy. These experts have been responsible for the education of speech therapists in ParkinsonNet and the development of the voice trainer. Coaching of speech therapists takes place in a group video call with the coach, speech therapist, and patient during three online treatment sessions (weeks 1, 3, and 5), in which the coach provides live feedback to the speech therapist.

Assessment procedures

Two assessors, who are kept blinded for the group allocation, will collect the data in the patient's home. Questionnaires are completed online by the patient within 3 days after the assessor's visit. Also at T0, patients receive an explanation on how to use the online platform and the Voice Trainer app. To use the Voice Trainer app and the online platform at the same time, patients need two devices (preferably a smartphone and a tablet or computer) (Fig. 2).

Outcome measures

Primary outcome measure

Our primary endpoint is quality of life using the summary index score of the Parkinson's Disease Questionnaire (PDQ-39) at 8 weeks follow-up (T1) [32, 33]. We have chosen the PDQ-39 as the primary outcome, because a positive impact of speech therapy on the lives of patients with PD is our highest goal. The PDQ-39 is a disease-specific measure of subjective health status and contains 39 questions, divided over eight important areas of health status: mobility, activities of daily living, emotional well-being, stigma, social support, cognitions, communication, and bodily discomfort. Patients indicate

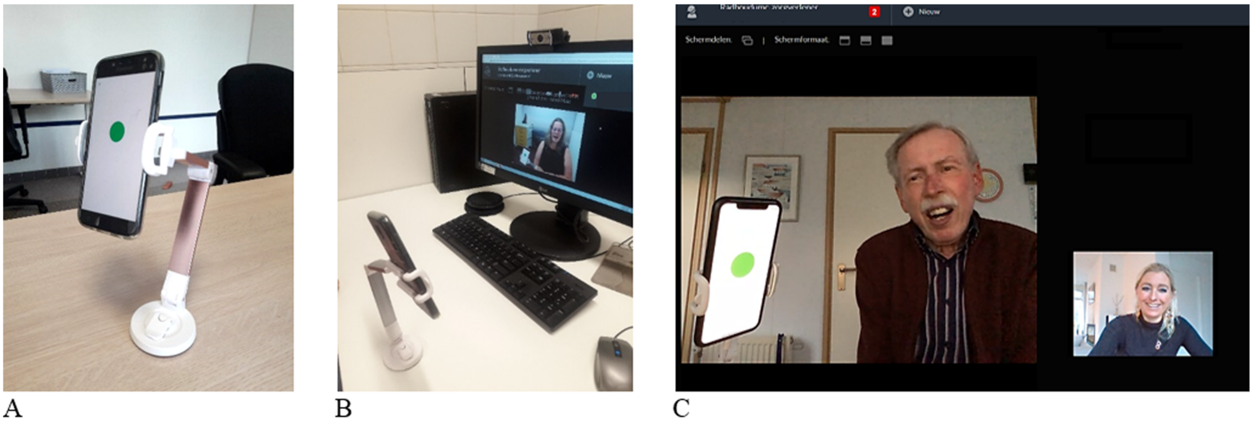

Fig. 2 Setup of using the Voice Trainer app and online platform. **A** Smartphone with voice trainer is correctly placed in a smartphone holder. **B** Smartphone is aimed at the camera of the second device (computer or tablet). **C** Voice trainer is visible for both patient and speech therapist on their computer screen

for every question how often they experience problems on a 5-point scale (never, seldom, sometimes, often, or always). A profile of scores can be calculated, indicating the impact of PD in the eight dimensions. The summary index score (SI) is calculated from the eight profile scores and provides an indication of the global impact of PD on health status. Studies evaluating the PDQ-39 show high levels of validity and reliability [32, 34]. The PDQ-39 is known as the most widely used PD-specific health-related quality of life questionnaire [35].

Secondary outcome measures

All secondary outcome measures are listed in Table 1 and include evaluation of speech and voice quality, voice handicap, speech intelligibility, severity of voice and speech complaints, mood and anxiety, health-related

Table 1 Schedule of measurements and outcome measures

Background variable	Instrument	Baseline	8 weeks	32 weeks[1]
Age, gender, disease duration, presence of advanced therapy	n.a.	√		
Cognitive functioning	Mini-Mental State Examination (MMSE) [36]	√		
	Category fluency task (animals) [37]	√		
Disease severity	Hoehn and Yahr stage [38]	√	√	√
	Total score on Movement Disorder Society - Unified Parkinson Disease Rating Scale (MDS-UPDRS) [39]	√	√	√
**Outcome measure**	**Instrument**			
Health-related quality of life	Parkinson's Disease Questionnaire (PDQ-39) [32–34]	√	√	√
	EuroQol-5D (EQ-5D) [40]	√	√	√
Speech quality	Radboud Dysarthria Assessment (RDA) [41]	√	√	√
Voice quality	Acoustic Voice Quality Index (AVQI) [42]	√	√	√
Voice handicap	Voice Handicap Index (VHI) [43]	√	√	√
Severity of voice and speech complaints, reported by patient	Radboud Oral Motor inventory for Parkinson's disease (ROMP) [44]	√	√	√
Severity of voice and speech complaints, reported by caregiver	Radboud Oral Motor inventory for Parkinson's disease (ROMP), adapted to caregiver	√	√	√
Speech intelligibility	Dutch intelligibility test – sentence level (NSVO-Z) [45]	√	√	√
Caregiver burden	Zarit caregiver Burden Interview Short Form (ZBI-12) [46]	√	√	√
Mood and anxiety	Hospital Anxiety and Depression Scale (HADS) [47]	√	√	√
Swallowing	Maximum swallowing speed (timed test) [48]	√	√	√

[1]T2 will only be performed in patients who were allocated to the intervention group

quality of life, and swallowing speed (the latter because of the possible impact of speech improvement on swallowing efficiency) [49]. Outcome measures at the caregiver level are caregiver burden, and caregiver reported severity of voice and speech complaints.

Background variables

Demographic data are collected at baseline and consist of age, gender, disease duration, and possible presence of advanced therapy, e.g., deep brain stimulation, subcutaneous apomorphine infusion, or levodopa-carbidopa intestinal gel. Patient's disease severity will be measured with the Movement Disorder Society – Unified Parkinson Disease Rating Scale (MDS-UPDRS [39];), including the Hoehn and Yahr scale. The Mini-Mental State Examination (MMSE [36];) and a category fluency task are used to assess general cognitive functioning. Demographic data, disease severity, and cognitive functioning will be used to describe the study population.

Sample size calculation

Based on a trial on the effectiveness of multidisciplinary care [50], we have calculated the sample size on our primary outcome with a conservative estimated PDQ-39 total score improvement of 2.5 points (SD = 5.8) in the intervention group, and no difference in controls. This estimated improvement exceeds the minimally important difference of 1.6 points [33]. A sample of 170 patients would be needed to show this expected difference. Allowing for 20% drop-out, we will include 215 patients.

Statistical analysis

Descriptive statistics

Means, standard deviations, and frequencies will be used to describe the outcome, background, and baseline variables.

Analysis effectiveness

Based on our hypothesis and design, two comparisons will be made: (1) the between-group differences at the primary endpoint (T1) and (2) the within-group differences in the experimental group after 24 weeks of follow-up (T1 versus T2). Statistical analyses will be performed based on the intention-to-treat principle. Analysis of covariance will be used with each of the outcomes as a dependent variable and group allocation as a fixed variable. Baseline values, age at baseline, Hoehn and Yahr stage, and disease duration will serve as covariates. We will also perform a process analysis and a planned post hoc analysis to identify which factors predict a successful outcome.

Data management and monitoring

The assessors will enter all data electronically in clinical data management system Castor EDC [24]. Participants will receive a unique consecutive personal identification code based on the order of enrollment. Original study forms will be entered and kept on file at the university medical center. Personal information about potential and enrolled participants is saved electronically on the server of the university medical center and is only accessible for the researchers. Personal data will not be shared. An independent data monitor will conduct three to four visits (after inclusion of the first three participants, during the inclusion period at least once a year, and after the follow-up measurement of the last participant) to randomly check the records for inaccuracies and errors in source data verification, serious adverse events, and inclusion and exclusion criteria. Furthermore, data collection program Castor EDC [24] keeps an audit trail log, which allows the data monitor to view all changes made to the study and reasons for the change. Since patients are at negligible risk during participation in this study, a data monitoring committee is not needed, and there is no ancillary and post-trial care for participants. In case of serious adverse events, the investigator will report them to the medical ethical committee without undue delay after obtaining knowledge of the events.

Dissemination and implementation

Trial results will be shared with the participating patients, speech therapists, and the general medical community by newsletters, with the Michael J Fox Foundation via a study report and with the academic community via a scientific paper. Additionally, trial findings will be shared with a relevant therapist via web-based communities for medical professionals specialized in Parkinson care in the Netherlands (www.ParkinsonConnect.nl) and with a wider audience via social media. The principles of speech treatment are generated from the guideline-based treatment that is considered as usual care, delivered by the speech therapist who is affiliated with the Dutch ParkinsonNet. However, the combination of the three specifications (personalized treatment, support by feedback app, and delivery of online treatment) is new and is studied here. Any results will be re-implemented via the continued education programs as delivered by the Dutch national ParkinsonNet approach.

Discussion

The PERSPECTIVE study is a large, well-designed, randomized controlled trial evaluating the effectiveness of speech therapy in persons living with PD. Particular strengths of our study include the randomized and

controlled design, the personalized treatment approach, the inclusion of PD patients irrespective of the disease stage, the long-term follow-up, the adequate power, and the choice for a patient-relevant primary endpoint.

We believe that the ultimate goal of any treatment for persons with PD is a relevant improvement of their quality of life. Intelligible speech is certainly thought to contribute to this, because we expect that patients are better able to handle communicative and social situations when their speech is more intelligible, which in turn will likely have a positive impact on quality of life. Obviously, there are many other factors that could affect the quality of life, like mobility, emotional well-being, or bodily discomfort. Taken together, choosing the quality of life as our primary endpoint is highly relevant for patients but also challenging at the same time, because it is only partially dependent on intelligibility. That is why we based our power calculation on finding a small, but clinically relevant improvement of quality of life after speech therapy.

The PERSPECTIVE study is a complex study, because we conduct our research in daily practice, with patients receiving the treatment within their own homes, instead of in a fully controlled situation within a hospital environment. While challenging, this remote home-based approach allows us to gain a realistic and generalizable view on the effectiveness of speech therapy in PD. We will discuss some of these challenges here.

The first challenge is that therapy is delivered by multiple speech therapists, which may be accompanied by inter-individual differences in treatment quality, which may in turn impact the results of the trial. In order to minimize this treatment variability, all speech therapists are being coached by an expert speech therapist for every single patient that is allocated to the experimental group. Moreover, all participating therapists are part of a professional network, which includes a thorough 3-day Parkinson-specific training course according to the latest guidelines prior to participation, as well as ascertainment of a high caseload in daily practice. In this way, we have taken specific measures that help to ascertain a delivery of high-quality treatment for every patient participating in the trial. An advantage is that we may be able to extrapolate the trial findings more readily to everyday clinical practice, as compared to studies that were performed on the more carefully controlled in clinic situations.

The second challenge is that we aim to include patients in all disease stages and with all grades of dysarthria severity, because we want to understand the effectiveness of the intervention for the full severity range, like it is presented in day-to-day clinical practice [51]. This leads to a heterogeneous study population, and therefore to more variation in the data, which could impact the power of our analysis. However, although the size of treatment results will differ between patients, we anticipate to show that dedicated speech therapy can make a difference for every PD patient with speech problems.

Although a non-inferiority trial has shown that telerehabilitation is feasible in patients with PD, also for speech therapy [17], this approach can still be a challenge for individual patients, e.g., due to a lack of technical skills, unstable internet connection, or cognitive problems, especially in advanced stages of PD. As a result, speech therapy could still be a challenge for individual patients and may lead to inclusion bias for our trial. However, we preferred online treatment over face-to-face treatment because of several advantages. First, online treatment reduces travel burden and costs and therefore saves patients' valuable time, energy, and expenses. Furthermore, online treatment has no limitations related to distance; as long as both patient and therapist have a stable internet connection, it does not matter where they are located. This is in particular relevant for patients who do not have physical access to a highly experienced speech therapist, which would make our remote speech therapy suitable for many patients, even for those living in remote areas. To overcome technical challenges and reduce potential inclusion bias, we will optimally support all participants by educating them as well as by testing a video call combined with the use of the voice trainer on the participants' own devices during the baseline home visit. We realize that this is not always feasible in daily practice, e.g., when there is too much travel distance between speech therapist and patient. However, based on the experiences from this trial, we expect to be able to give specific recommendations on how to use the setup and to draw conclusions about what type of patients will be able to use remote speech therapy, and what sort of external support would be needed for this.

One of the limitations of a non-pharmacological trial is that blinding is a challenge. The assessor may notice an improvement in speech at the T1 measurement compared to the baseline measurement. Also, it is possible that patients or speech therapists accidentally reveal their group allocation (experimental or control group) to the assessor. To minimize this risk, patients and speech therapists are repeatedly requested to keep their group allocation secret to the assessor. We will assess the success of this blinding procedure after the trial by debriefing both the participants and the assessors.

Finally, we would like to mention that the trial will be conducted in part during the COVID-19 pandemic. Therefore, home-based measurements were not allowed during a few months when we started the study. We decided to proceed with the planned T1 and T2

Table 2 Trial registration data set

Data category	Information
Primary registry and trial identifying number	ClinicalTrials.gov NCT03963388
Date of registration in primary registry	May 24 2019
Secondary identifying numbers	NL67867.091.18
Source(s) of monetary or material support	Michael J Fox Foundation for Parkinson's Research
Public title	PERsonalized SPEeCh Therapy for actIVE Conversation
Scientific title	The PERSPECTIVE Study: PERsonalized SPEeCh Therapy for actIVE Conversation
Countries of recruitment	The Netherlands
Health condition(s) or problem(s) studied	Parkinson's disease, speech problems
Interventions	Online speech therapy, delivered by specialized speech therapists. Speech therapy will be complemented by a real-time visual feedback app (the Voice Trainer app).
Key inclusion and exclusion criteria	Inclusion criteria: A diagnosis of idiopathic PD, problems in intelligibility affecting daily communication (as indicated by the patient and/or the caregiver), a desire for improvement, willing and able to receive online treatment.
	Exlusion criteria: Recent (<1 year) speech therapy, voice or speech problems due to other causes, communication difficulties based on language problems without predominantly reduced intelligibility, inability to receive online treatment.
Study type	Interventional
	Allocation: randomized intervention model. Single blinded (outcomes assessor).
Date of first enrolment	March 2019
Target sample size	215
Recruitment status	Recruiting
Primary outcome(s)	Total score of the Parkinson's Disease Questionnaire (PDQ-39)
Key secondary outcomes	Radboud Dysarthria Assessment (RDA)<br>• Speech quality [Time Frame: Baseline (T0), primary endpoint after 8 weeks (T1), follow-up after 32 weeks (T2)]
	Acoustic Voice Quality Index (AVQI)<br>• Voice quality [Time Frame: Baseline (T0), primary endpoint after 8 weeks (T1), follow-up after 32 weeks (T2)]
	Voice Handicap Index (VHI)<br>• Voice handicap [Time Frame: Baseline (T0), primary endpoint after 8 weeks (T1), follow-up after 32 weeks (T2)]
	Radboud Oral Motor inventory for Parkinson's disease (ROMP)<br>• Severity of voice and speech complaints, reported by patient [Time Frame: Baseline (T0), primary endpoint after 8 weeks (T1), follow-up after 32 weeks (T2)]
	Radboud Oral Motor inventory for Parkinson's disease (ROMP), adapted to caregiver<br>• Severity of voice and speech complaints, reported by caregiver [Time Frame: Baseline (T0), primary endpoint after 8 weeks (T1), follow-up after 32 weeks (T2)]
	Dutch intelligibility test - sentence level (NSVO-Z)<br>• Speech intelligibility [Time Frame: Baseline (T0), primary endpoint after 8 weeks (T1), follow-up after 32 weeks (T2)]
	Zarit caregiver Burden Interview Short Form (ZBI-12)<br>• Caregiver burden [Time Frame: Baseline (T0), primary endpoint after 8 weeks (T1), follow-up after 32 weeks (T2)]
	Hospital Anxiety and Depression Scale (HADS)<br>• Mood and anxiety [Time Frame: Baseline (T0), primary endpoint after 8 weeks (T1), follow-up after 32 weeks (T2). Minimum score = 0 (no anxiety or depression), maximum score = 42 (most anxiety or depression).]
	EuroQol-5D (EQ-5D)<br>• Health-related quality of life [Time Frame: Baseline (T0), primary endpoint after 8 weeks (T1), follow-up after 32 weeks (T2)]
	Maximum swallowing speed (timed test)<br>• Swallowing [Time Frame: Baseline (T0), primary endpoint after 8 weeks (T1), follow-up after 32 weeks (T2)]

measurements using online measurements. No new patients were included during lock-down. This strategy has resulted in missing data for several secondary outcome measures, e.g., voice and speech quality. However, our primary outcome and other surveys could be collected as planned without any problems. Importantly, a great advantage of the remote therapy that we study here is that all treatments could be continued online, making our approach future-proof.

Trial status

The inclusion started in March 2019 and was finalized in March 2021. Follow-up measurements started in May 2019 and will be finalized in November 2021 (Table 2).

Acknowledgements
This study is made possible by a grant of the Michael J. Fox Foundation.

Trial sponsor
Radboud University Medical Center
Contact name: Dr. Hanneke Kalf
PO Box 9101, 6500 HB Nijmegen

hanneke.kalf@radboudumc.nl
Telephone: 024-3668185

Protocol version

Issue date: March 31 2021
Protocol amendment number: 2
Authors: Dr. N.M. de Vries, Dr. B.R. Bloem, Dr. J.G. Kalf, and J.J.L. Maas
Revision chronology: 2019-Jan-7 original
2019-Feb-28 Amendment 01: Primary reason for amendment: Addition of treatment report in which speech therapists will report treatment intensity and if the patient's personal speech goal is reached.
2019-Apr-17 Amendment 02: Primary reason for amendment: Coaching of the speech therapist is added to the protocol.

Authors' contributions

B.R. Bloem, N.M. de Vries, and J.G. Kalf conceived the study and initiated the study design. J.J.L. Maas helped with the implementation. All authors contributed to the refinement of the study protocol and approved the final manuscript.

Funding

Dr. De Vries had received research grants from the Netherlands Organization of Health Research and Development. Prof. Bloem currently serves as an Editor-in-Chief for the *Journal of Parkinson's Disease*; serves on the editorial of Practical Neurology and Digital Biomarkers; has received honoraria from serving on the scientific advisory board for Abbvie, Biogen, and UCB; has received fees for speaking at conferences from AbbVie, Zambon, Roche, GE Healthcare, and Bial; and has received research support from the Netherlands Organization for Scientific Research, the Michael J Fox Foundation, UCB, Abbvie, the Stichting Parkinson Fonds, the Hersenstichting Nederland, the Parkinson's Foundation, Verily Life Sciences, Horizon 2020, and the Parkinson Vereniging.

Availability of data and materials

Access to the data is restricted, meaning that researchers who are interested in the re-use of the data are asked to contact the researchers for permission. Approval is given after a signed agreement.

Declarations

Ethics approval and consent to participate

Full ethical approval has been granted by the medical ethical committee of Arnhem-Nijmegen (NL67867.091.18), and the PERSPECTIVE trial is registered at ClinicalTrials.gov (NCT03963388) on May 24, 2019.

Consent for publication

All authors have provided consent for publication.

Competing interests

The authors declare that they have no competing interests.

Author details

[1]Donders Institute for Brain, Cognition and Behaviour, Department of Neurology, Centre of Expertise for Parkinson & Movement Disorders, Radboud University Medical Centre, Nijmegen, The Netherlands. [2]Donders Institute for Brain, Cognition and Behaviour, Department of Rehabilitation, Radboud University Medical Centre, Nijmegen, The Netherlands.

Received: 18 May 2021 Accepted: 7 March 2022
Published online: 08 April 2022

References

1. Miller N, Allcock L, Jones D, Noble E, Hildreth AJ, Burn DJ. Prevalence and pattern of perceived intelligibility changes in Parkinson's disease. J Neurol Neurosurg Psychiatry. 2007;78(11):1188–90. https://doi.org/10.1136/jnnp.2006.110171.
2. Miller N, Noble E, Jones D, Burn D. Life with communication changes in Parkinson's disease. Age Ageing. 2006;35(3):235–9. https://doi.org/10.1093/ageing/afj053.
3. Maillet A, Krainik A, Debu B, Tropres I, Lagrange C, Thobois S, et al. Levodopa effects on hand and speech movements in patients with Parkinson's disease: a FMRI study. PLoS One. 2012;7(10):e46541. https://doi.org/10.1371/journal.pone.0046541.
4. Pinto S, Ozsancak C, Tripoliti E, Thobois S, Limousin-Dowsey P, Auzou P. Treatments for dysarthria in Parkinson's disease. Lancet Neurol. 2004;3(9):547–56. https://doi.org/10.1016/S1474-4422(04)00854-3.
5. Atkinson-Clement C, Sadat J, Pinto S. Behavioral treatments for speech in Parkinson's disease: meta-analyses and review of the literature. Neurodegener Dis Manag. 2015;5(3):233–48. https://doi.org/10.2217/nmt.15.16.
6. Herd CP, Tomlinson CL, Deane KH, Brady MC, Smith CH, Sackley CM, et al. Speech and language therapy versus placebo or no intervention for speech problems in Parkinson's disease. Cochrane Database Syst Rev. 2012;(8):Cd002812.
7. Herd CP, Tomlinson CL, Deane KHO, Brady MC, Smith CH, Sackley CM, et al. Comparison of speech and language therapy techniques for speech problems in Parkinson's disease. Cochrane Database Syst Rev. 2012;8. https://doi.org/10.1002/14651858.CD002814.pub2.
8. Sackley CM, Smith CH, Rick C, Brady MC, Ives N, Patel R, et al. Lee Silverman voice treatment versus standard NHS speech and language therapy versus control in Parkinson's disease (PD COMM pilot): study protocol for a randomized controlled trial. Trials. 2014;15(1):213. https://doi.org/10.1186/1745-6215-15-213.
9. Ramig LA, Fox CM, Halpern AE, Spielman JL, Freeman K. Randomized clinical trial (RCT) of speech and voice treatment for Parkinson's disease [abstract]. Stockholm: Movement Disorders; 2014.
10. Ramig L, Halpern A, Spielman J, Fox C, Freeman K. Speech treatment in Parkinson's disease: randomized controlled trial (RCT). Mov Disord. 2018;0(0).
11. Ramig LO, Sapir S, Fox C, Countryman S. Changes in vocal loudness following intensive voice treatment (LSVT(R)) in individuals with Parkinson's disease: a comparison with untreated patients and normal age-matched controls. Mov Disord. 2001;16(1):79–83. https://doi.org/10.1002/1531-8257(200101)16:1<79::AID-MDS1013>3.0.CO;2-H.
12. Mahler LA, Ramig LO, Fox C. Evidence-based treatment of voice and speech disorders in Parkinson disease. Curr Opin Otolaryngol Head Neck Surg. 2015;23(3):209–15. https://doi.org/10.1097/MOO.0000000000000151.
13. de Swart BJM, Willemse SC, Maassen BAM, Horstink MWIM. Improvement of voicing in patients with Parkinson's disease by speech therapy. Neurology. 2003;60(3):498–500. https://doi.org/10.1212/01.WNL.0000044480.95458.56.
14. Levitt JS, Chitnis S, Walker-Batson D. The effects of the "SPEAK OUT! (R)" and "LOUD Crowd (R)" voice programs for Parkinson disease. Int J Health Sci. 2015;3(2):13–9.
15. Wight S, Miller N. Lee Silverman Voice Treatment for people with Parkinson's: audit of outcomes in a routine clinic. Int J Lang Commun Disord. 2015;50(2):215–25. https://doi.org/10.1111/1460-6984.12132.
16. Dias AE, Limongi JCP, Barbosa ER, Hsing WT. Voice telerehabilitation in Parkinson's disease. CODAS. 2016;28(2):176–81. https://doi.org/10.1590/2317-1782/20162015161.
17. Theodoros DG, Hill AJ, Russell TG. Clinical and quality of life outcomes of speech treatment for Parkinson's disease delivered to the home via telerehabilitation: a noninferiority randomized controlled trial. Am J Speech Lang Pathol. 2016;25(2):214–32. https://doi.org/10.1044/2015_AJSLP-15-0005.
18. Ho AK, Iansek R, Bradshaw JL. The effect of a concurrent task on Parkinsonian speech. J Clin Exp Neuropsychol. 2002;24(1):36–47. https://doi.org/10.1076/jcen.24.1.36.972.
19. Radboudumc. Voice trainer: Radboudumc; 2019 Available from: https://www.radboudumc.nl/afdelingen/revalidatie/voice-trainer.
20. de Swart BJM, de Wit S, Kalf H. De Pitch Limiting Voice Treatment. Handleiding voor de behandeling hypokinetische dysartrie. Nijmegen: Radboudumc, afdeling Revalidatie; 2016.
21. Kalf H, de Swart BJM, Bonnier-Baars M, Kanters J, Hofman M, Kocken J, et al. Logopedie bij de ziekte van Parkinsons: een richtlijn van de Nederlandse Vereniging voor Logopedie en Foniatrie. Woerden: Nederlandse Vereniging voor Logopedie en Foniatrie; 2008.
22. Bloem BR, Rompen L, Vries NM, Klink A, Munneke M, Jeurissen P. ParkinsonNet: a low-cost health care innovation with a systems approach from The Netherlands. Health Aff (Millwood). 2017;36(11):1987–96. https://doi.org/10.1377/hlthaff.2017.0832.
23. Bloem BR, Munneke M. Revolutionising management of chronic disease: the ParkinsonNet approach. BMJ. 2014;348:g1838.
24. BV C. Castor Electronic Data Capture. Amsterdam. 2016.
25. Postuma RB, Berg D, Stern M, Poewe W, Olanow CW, Oertel W, et al. MDS clinical diagnostic criteria for Parkinson's disease. Mov Disord. 2015;30(12):1591–601. https://doi.org/10.1002/mds.26424.

26. Kalf JG, De Swart BJM, Bonnier-Baars M, Hofman M, Kanters J, Kocken J, et al. Guidelines for speech-language therapy in Parkinson's disease. Nijmegen, The Netherlands/Miami (FL), U.S.A.: Lemma Publishers; 2011.
27. Sturkenboom IH, Graff MJ, Borm GF, Veenhuizen Y, Bloem BR, Munneke M, et al. The impact of occupational therapy in Parkinson's disease: a randomized controlled feasibility study. Clin Rehabil. 2013;27(2):99–112. https://doi.org/10.1177/0269215512448382.
28. Sturkenboom IHWM, Graff MJL, Hendriks JCM, Veenhuizen Y, Munneke M, Bloem BR, et al. Efficacy of occupational therapy for patients with Parkinson's disease: a randomised controlled trial. Lancet Neurol. 2014;13(6): 557–66. https://doi.org/10.1016/S1474-4422(14)70055-9.
29. Ginis P, Nackaerts E, Nieuwboer A, Heremans E. Cueing for people with Parkinson's disease with freezing of gait: a narrative review of the state-of-the-art and novel perspectives. Ann Phys Rehabil Med. 2017;61(6):407–13. https://doi.org/10.1016/j.rehab.2017.08.002.
30. De Monchy N. Een app ter ondersteuning van logopedische behandeling. Parkinson Magazine. 2015;1:14–5.
31. Kalf H, van Zundert S, Scholten S, van Dongen-Stiemer F, Donkers A, Wassenaar-Dam C, et al. Logopedie bij de ziekte van Parkinson, een herziene richtlijn van de Nederlandse Vereniging voor Logopedie en Foniatrie. Woerden: Nederlandse Vereniging voor Logopedie en Foniatrie; 2017.
32. Jenkinson C, Fitzpatrick R, Peto V, Greenhall R, Hyman N. The Parkinson's Disease Questionnaire (PDQ-39): development and validation of a Parkinson's disease summary index score. Age Ageing. 1997;26(5):353–7. https://doi.org/10.1093/ageing/26.5.353.
33. Peto V, Jenkinson C, Fitzpatrick R. Determining minimally important differences for the PDQ-39 Parkinson's disease questionnaire. Age Ageing. 2001;30(4):299–302. https://doi.org/10.1093/ageing/30.4.299.
34. Peto V, Jenkinson C, Fitzpatrick R, Greenhall R. The development and validation of a short measure of functioning and well being for individuals with Parkinson's disease. Qual Life Res. 1995;4(3):241–8.
35. Chen K, Yang YJ, Liu FT, Li DK, Bu LL, Yang K, et al. Evaluation of PDQ-8 and its relationship with PDQ-39 in China: a three-year longitudinal study. Health Qual Life Outcomes. 2017;15(1):170. https://doi.org/10.1186/s12955-017-0742-5.
36. Folstein MF, Folstein SE, McHugh PR. "Mini-mental state": a practical method for grading the cognitive state of patients for the clinician. J Psychiatr Res. 1975;12(3):189–98. https://doi.org/10.1016/0022-3956(75)90026-6.
37. Mirandez RM, Aprahamian I, Talib LL, Forlenza OV, Radanovic M. Multiple category verbal fluency in mild cognitive impairment and correlation with CSF biomarkers for Alzheimer's disease. Int Psychogeriatr. 2017;29(6):949–58. https://doi.org/10.1017/S1041610217000102.
38. Hoehn MM, Yahr MD. Parkinsonism. onset, progression, and mortality. 1967; 17(5):427.
39. Goetz CG, Tilley BC, Shaftman SR, Stebbins GT, Fahn S, Martinez-Martin P, et al. Movement Disorder Society-sponsored revision of the Unified Parkinson's Disease Rating Scale (MDS-UPDRS): scale presentation and clinimetric testing results. Mov Disord. 2008;23(15):2129–70.
40. Balestroni G, Bertolotti G. EuroQol-5D (EQ-5D): an instrument for measuring quality of life. Monaldi Arch Chest Dis. 2012;78(3):155–9.
41. Knuijt S, Kalf JG, van Engelen BGM, de Swart BJM, Geurts ACH. The Radboud Dysarthria Assessment: development and clinimetric evaluation. Folia Phoniatr Logop. 2017;69(4):143–53.
42. Maryn Y, De Bodt M, Roy N. The Acoustic Voice Quality Index: toward improved treatment outcomes assessment in voice disorders. J Commun Disord. 2010;43(3):161–74. https://doi.org/10.1016/j.jcomdis.2009.12.004.
43. Jacobson BH, Johnson A, Grywalski C, Silbergleit A, Jacobson G, Benninger MS, et al. The Voice Handicap Index (VHI). Am J Speech Lang Pathol. 1997; 6(3):66–70. https://doi.org/10.1044/1058-0360.0603.66.
44. Kalf JG, Borm GF, de Swart BJ, Bloem BR, Zwarts MJ, Munneke M. Reproducibility and validity of patient-rated assessment of speech, swallowing, and saliva control in Parkinson's disease. Arch Phys Med Rehabil. 2011;92(7):1152–8. https://doi.org/10.1016/j.apmr.2011.02.011.
45. Martens H, Nuffelen G, Bodt M. Nederlandstalig Spraakverstaanbaarheidsonderzoek - Zinsniveau (NSVO-Z). In: Dutch Sentence Intelligibility Assessment (DSIA); 2010.
46. Higginson IJ, Gao W, Jackson D, Murray J, Harding R. Short-form Zarit Caregiver Burden Interviews were valid in advanced conditions. J Clin Epidemiol. 2010;63(5):535–42. https://doi.org/10.1016/j.jclinepi.2009.06.014.
47. Herrmann C. International experiences with the Hospital Anxiety and Depression Scale-a review of validation data and clinical results. J Psychosom Res. 1997;42(1):17–41. https://doi.org/10.1016/S0022-3999(96)00216-4.
48. Kanna SV, Bhanu K. A simple bedside test to assess the swallowing dysfunction in Parkinson's disease. Ann Indian Acad Neurol. 2014;17(1):62–5. https://doi.org/10.4103/0972-2327.128556.
49. El Sharkawi A, Ramig L, Logemann JA, Pauloski BR, Rademaker AW, Smith CH, et al. Swallowing and voice effects of Lee Silverman Voice Treatment (LSVT): a pilot study. J Neurol Neurosurg Psychiatry. 2002;72(1):31–6. https://doi.org/10.1136/jnnp.72.1.31.
50. Marck MA, Bloem BR, Borm GF, Overeem S, Munneke M, Guttman M. Effectiveness of multidisciplinary care for Parkinson's disease: a randomized, controlled trial. Mov Disord. 2013;28(5):605–11. https://doi.org/10.1002/mds.25194.
51. J. Holmes R, M. Oates J, J. Phyland D, J. Hughes A. Voice characteristics in the progression of Parkinson's disease. Int J Lang Commun Disord. 2000; 35(3):407–18. https://doi.org/10.1080/136828200410654.

Publisher's Note

Springer Nature remains neutral with regard to jurisdictional claims in published maps and institutional affiliations.

Ready to submit your research? Choose BMC and benefit from:

- fast, convenient online submission
- thorough peer review by experienced researchers in your field
- rapid publication on acceptance
- support for research data, including large and complex data types
- gold Open Access which fosters wider collaboration and increased citations
- maximum visibility for your research: over 100M website views per year

At BMC, research is always in progress.

Learn more biomedcentral.com/submissions

BMC
